# Music-based interventions for people with profound and multiple learning disabilities: A systematic review of the literature

**DOI:** 10.1177/17446295221087563

**Published:** 2022-04-29

**Authors:** Rosie Rushton, Lila Kossyvaki, Emmanouela Terlektsi

**Affiliations:** 1724 University of Birmingham, Department of Disability Inclusion and Special Needs (DISN), School of Education, Birmingham, UK

**Keywords:** interventions, music, profound intellectual/learning disabilities, review

## Abstract

Music is motivational, accessible and engaging for individuals with learning disabilities. Several systematic reviews have addressed the effects of music activity on people with learning disabilities; however, none has specifically reviewed the use of musical activity with people with profound and multiple learning disabilities.

This review aimed to: 1. identify peer-reviewed studies and describe the characteristics of evidence-based musical activity used with people with profound and multiple learning disabilities and 2. evaluate and analyse the effectiveness of these music-based interventions and identify gaps within current research. A systematic search was conducted in April 2021 identifying seven peer-reviewed studies which included music-based interventions with at least one person with profound and multiple learning disabilities.

Findings reveal the interventions varied in their frequency, duration and content. The outcome of most (n=6) interventions documented the development of the participants’ social skillset. Interventions were predominantly (n=6) implemented by facilitators with musical expertise. The diverse and novel nature of the reviewed studies highlights a need to expand and enhance research with this population.

## Introduction

Music is easily accessible, highly motivational and can be used as an effective tool to support wellbeing, mood regulation and social interactions (Hallam and MacDonald, 2013; Murphy and McFerran, 2017). From the early stages of development, music is recorded to affect the emotional state of its listener (Juslin and Sloboda, 2013). Entwined within everyday life, throughout human societies, parents employ musical interactions to support communication and bonding with their child (Custodero and Johnson-Green, 2003; Zeedyk, 2008).

Music has been recorded as highly motivational for young people with profound and multiple learning disabilities (Corke, 2012; Ockelford, 2008). A frequent activity within both their home (Axelsson and Wilder, 2014) and school lives (Maes et al., 2010), music is reported to affect the mood, communication, and playfulness of people with profound and multiple learning disabilities (Rushton and Kossyvaki, 2020, 2022). Whilst there is not a universally agreed definition of the characteristics of people with profound and multiple learning disabilities (Nind and Strnadová, 2020), this heterogenous population have a profound intellectual disability often combined with physical disabilities, sensory impairments and complex medical needs (Doukas et al., 2017). Other terms adopted to identify this population within literature include; people with profound intellectual and multiple disabilities (PMLD), profound and multiple intellectual disabilities (PMID), or individuals with complex and/or profound needs. Within the United Kingdom the term profound and multiple learning disabilities has been widely accepted within education, healthcare and academic settings and the description provided by Doukas et al. (2017) is adopted within this study.

Music activity for people with profound and multiple learning disabilities can be broadly divided into: 1. musical education, in which the purpose of music is to teach musical skill (Ockelford, 2008); 2. music therapy in which a trained professional music-therapist uses music as a vehicle for therapeutic benefits (Alder and Samsonova-Jellison, 2017; Thompson, 2019); and 3. social music, in which music is used to support the social interaction and behaviours between people with profound and multiple learning disabilities, their peers, and supporting adults, in both educational and recreational contexts (Corke, 2012; Ramney, 2011). Although there may be crossovers between the techniques used by practitioners across the above-mentioned musical activities, these are largely defined by their intended outcomes and the individual/s facilitating the musical activity.

The benefits of music for people with profound and multiple learning disabilities are often founded on literature which does not specifically include people with profound and multiple learning disabilities. Literature including typically developing babies (Ockelford, 2000; Stern, 2010), or disabled populations with higher cognitive abilities are used to advocate for the use of music with people with profound and multiple learning disabilities. However, the usefulness and transferability of these findings may be limited (Adler & Samsonova-Jellison, 2017), as people with profound and multiple learning disabilities often experience the world in unique and unconventional ways (Grace, 2017).

Though there have been several systematic reviews focusing on the use of music (Brown and Jellison, 2012; Hooper et al., 2008; Rolka and Silverman, 2015) and music therapy (DeVries et al., 2015; James et al., 2015; Mayer-Benarous et al., 2021) for people with learning disabilities, these papers do not review the literature specifically for people with profound and multiple learning disabilities. Therefore, the uniqueness of this review is its focus on the use of music, in its educational, therapeutic and social forms, as an intervention specifically for people with profound and multiple learning disabilities.

The aims of the review were the following:

- to identify peer-reviewed studies and describe the characteristics of the musical activity used with people with profound and multiple learning disabilities;
- to evaluate and analyse the effectiveness of these music-based interventions; and
- to identify gaps within current research and suggest recommendations for future research and practice.

The authors approach this review through the biopsychosocial model of disability (Engel, 1977). This model recognises the complex medical needs of people with profound and multiple learning disabilities, which often require ongoing medical treatment, to support quality of, and in some instances sustain life (Rees, 2017), while acknowledging the further compounding disabling barriers present within society. It sits in-between the medical and social model of disability. The former conceives that since disabilities are diseases or illnesses, disabled people require methods of treatment to ‘repair’ their biological state (Thompson, 2019), while the latter model assumes that disability is a result of social and political constructs, disabling barriers and lack of acceptance within wider society (Barnes, 2019). Considering music interventions, people with profound and multiple learning disabilities are likely to have some biological and physiological disabilities, which may impact on their physical functioning, communication methods, and the way they may access music. At the same time the environment, materials, and approaches of others (adults and peers) can be altered, to create a more accessible and meaningful intervention.

## Methodology

### Search Procedure

The first author conducted a systematic search of two electronic databases which are commonly used to conduct a search of educational studies, ProQuest and Education Resources Information Center (ERIC), in April 2021. The title and the abstracts of the identified papers were considered. A hand search of the selected papers’ reference lists was also implemented to ensure that there were no omissions of related papers. The search terms used were “music” OR “musical” AND “profound disabilit*” OR “profound intellectual disability” OR “profound learning disabilit*” OR “profound and multiple learning disabilit*” OR “complex needs”. The timeframe for studies to be included within the review was January 2010–April 2021. This timeframe was selected to review recent studies and to contribute to the up-to-date knowledge within the field. Furthermore, within this timeframe there were likely to be fewer inconsistencies in the terminology used to describe this specific population (Bellamy et al., 2010). This terminology, along with the expected life experiences and acknowledgement of people with profound and multiple learning disabilities, has developed significantly in the global north over the past thirty years (Nind and Strnadová, 2020). Inconsistencies in ambiguous terminology, such as ‘high support needs’, ‘people with additional or complex needs’, ‘the most severely disabled’, has led to a lack of cohesive representation of this population in education, research and policy making (Carnaby, 2004; PMLD Network, 2002).

### Inclusion and exclusion criteria

The inclusion criteria were developed to support the focus of this review, music-based interventions with people with profound and multiple learning disabilities. They were designed to be practical and manageable (Xiao and Watson, 2019). Inclusion criteria for each reviewed study were the following (see Table 1).

**Table 1.** Inclusion Criteria.

Papers had to:
1. be published in a peer-reviewed journal
2. be published between 2010 and 2020
3. be written in English
4. involve at least one participant with profound and multiple learning disabilities, or profound and multiple intellectual disabilities as defined by Doukas et al. (2017) of all ages
5. include empirical data
6. use an intervention, for which music is a key feature, as the independent variable
7. consider an intervention that has been used on more than one occasion.

Some papers were rejected because of the following exclusion criteria (see Table 2):

**Table 2.** Exclusion Criteria.

Papers did not:
1. use music as a key feature of the intervention but rather as a secondary component
2. clearly identify a participant/s as having profound and multiple learning disabilities or the definitions they used were different to the one adopted in this study
3. include empirical data (e.g. were review or opinion papers, theoretical books/book chapters)
4. include those published in non-peer-reviewed/professional journals or conference proceedings.

**Figure 1.**
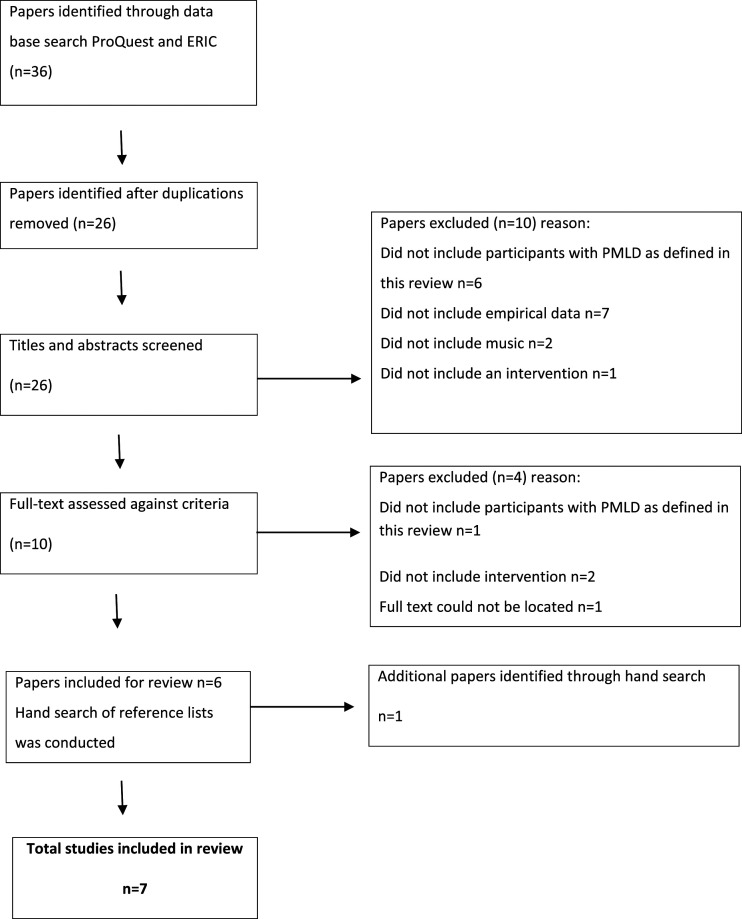
Process of literature search (adapted from Xiao and Watson, 2019).

The first author removed the duplications and considered the titles and abstracts of all collected papers. From this process, ten potential papers were identified. After accessing the full text, three papers were rejected as they did not meet the inclusion criteria. A total number of seven papers, meeting the inclusion criteria above, are included within this review (see Figure 1). The full text of these seven papers was also checked by the second author who confirmed that they meet the inclusion criteria.

Data extraction:

Each study was reviewed with the aim to extract the following data:

*Participant characteristics*: i. age, ii. gender and iii) description of disability/diagnosis.

*Use of music*: i. *education*, ii, *social,* iii) *therapy.* Musical activity was defined as *music education* if the primary purpose of using music was to teach musical skills. Music activity was defined as *social music* when used to support the social interactions and behaviours between people with profound and multiple learning disabilities, their peers, and supporting adults, and *music therapy* when delivered by a trained music therapist.

*Type of music*: i. *live,* in which either the facilitator or the participant/s used live musical sounds or ii. *pre-recorded,* in which either the facilitator or the participant/s accessed pre-recorded musical content as part of the intervention.

*Implementation of intervention*: i. frequency, ii. duration, iii. equipment needed, iv. facilitator, v. setting, vi. intended outcomes and vii. reported effectiveness of intervention.

A summary of the above is presented in Table 3.

**Table 3.** Included studies.

	*Participant information*			*Musical qualities*			*Intervention*							
References*	Sex	Age	Description of diagnosis	Musical Activity	Type of music (live/pre-recorded)	Musical elements	Frequency	Duration of session	Equipment or materials	Facilitator	Setting	Measured Outcomes	Reported effectiveness	Total robustness scores** /30
Bosch et al. (2017)	Unknown Total participants n=13 With PMLD n=2	18+	Profound intellectual disability	Social music	Pre-recorded soundscapes	Listening to a range of pre-recorded soundscapes	10 separate days over a course of 9 weeks	Up to 20 minutes per session	Six audio speakers	Direct support professional	Day care service centre Dedicated room	Mood/behaviours **Tool:** Simplified version of the Assessment Auditory Environment (AAE, van Den Bosch et al., 2016)	**Positive effect.** Reduction in distress behaviours	23
Fenech (2015)	5 males 9 females	22–74 years	Profound levels of neurological disability	Social music	Live and pre-recorded	Joining in with simple musical instruments Dancing Humming	Unknown	45 minutes per condition	Pre-recorded music Simple musical instruments	Volunteer DJ	Big public space in residential care setting.	Engagement **Tool:** Individual Child Engagement Record (ICER; Kishida and Kemp, 2006)	**Positive effect.** Higher levels of engagement.	18
Kossyvaki and Curran (2020)	Total participants n=5 4 males 1 female With PMLD 1 male	5 years	Autism and profound intellectual disability	Social music	Pre-recorded	Interaction with a pre-recorded interactive digital interface Improvisation Composition Orchestration Imitation	Twice weekly sessions over a 5-week period	12 minutes	‘Cosmo hardware’ a set of six auditory and visual switches	Researcher with music teaching experience Member of class staff	Special School Setting small room within the classroom	Engagement and communication **Tool:** The Engagement and Social Communication Checklist. Developed by the researchers.	**Mixed results** Some positive effects, increase in requests, anticipation. Fluctuating levels of engagement.	24
Ockelford et al. (2011)	Total participants with PMLD n=20 Gender unknown	11 years 11 months-17 years 7 months	Young people with Profound and multiple learning disabilities. Profound levels of global developmental delay, non-verbal, majority wheelchair users, many some degree of visual impairment.	Music Education	Live	All Join in Framework Introductory song, other songs including specific musical elements, concluding songs.	Weekly (with breaks for half-terms) 24 sessions between January and July.	45 minutes weekly	Untuned percussion instruments and electric keyboard	Special School music teacher & first-author	Special School Pupils classroom	Musical reactivity, proactivity, and interactivity **Tool:** Sounds of Intent framework (Ockelford, 2000)	**Positive effect.** Marked rate of musical development.	21
Rushton and Kossyvaki (2020)	4 males 1 female	8–10 years	Profound and multiple learning disabilities. Complex needs Non-ambulatory	Social Music	Live & pre-recorded	Pre-recorded ‘playful’ music. Musical Play intervention Imitation Playful, non-conventional use of instruments	Weekly over a 5-week period	15–35 minutes (25 min average)	Playful pre-recorded music wrist bells, wrist shell-shakers, chime-tree, flat-skinned drum, and large shell-shakers	Class staff Music specialist	Special School Music or playroom.	Frequency of reactive and reciprocal play behaviours. **Tool:** Adapted version of the Social Play Record (White, 2006).	**Positive effect.** Increase in frequency of play-skill behaviours in relation to cognitive ability.	20
Thompson and McFerran (2015)	3 females 1 male	10–15 years	IQ <50 Cerebral palsy and intellectual disability	Music therapy	1:1 live music therapy sessions	Improvisation Tempo/volume changes Anticipation Participant preferences	22 sessions over a period of 6 months	30 min Twice per week	Range of different instruments	Music therapist	Special School	Frequency of communicative behaviours **Tool:** Adapted version of Inventory of potentially communicative acts (IPCA, Sigafoos et al., 2006)	**No effect.** No different than control measure (toy play)	21
Wilson and MacDonald (2019)	Total participants n=37 12 females 25 males With PMLD n=unknown	18+	Profound learning difficulties	Social music	Live	Group music exercises, Instrumental training, composing, rehearsing, performing	Weekly sessions over a period of 10 weeks	2 hours per week	Djembes Boom Whackers Keyboards	Limelight Music (music education charity)	Community rehearsal facility and resource centre.	Social interaction and relationship skills. Behaviour	**Positive effect.** Less frequent repetitive behaviours, fewer instances of fingers being placed in ears. Increased engagement and attentiveness.	13

*In alphabetical order

**first author’s scores

The robustness and quality of each study was evaluated using the ‘Matrix table to derive confidence in the robustness of empirical studies’, developed by Terlektsi et al. (2019). Although the matrix was originally developed for use with interventions in the field of deafness, it was felt that the rigour of the tool and the descriptors it provides were appropriate and transferable to the studies reviewed within this paper. Furthermore, to the authors’ knowledge, there is no tool developed or available for evaluating interventions specifically for people with profound and multiple learning disabilities. The framework gives the following ten quality indicators a score of 1, 2 or 3 depending on evidence of impact, a score of one being the least, and three the most.

The assessed quality indicators were the following: i. objectives, ii. outcome measures, iii. research design, iv. intervention, v. implications for practice, vi. sample size, vii. generalisability, viii. data reporting, ix. critical reflections of limitations of study and x. reporting of evaluation. For each reviewed study, the overall minimum score was 10 and the maximum 30. The first author reviewed and scored all papers. To reduce bias and establish inter-rater reliability, the papers were then reviewed by a second reviewer (the second or the third author) independently. Where the total score for both reviewers fell between the same range (e.g., between 10 and 19 indicating impressionistic or 20 and 30 indicating moderate to strong robustness) an agreement was recorded. For papers for which there would be a disagreement, the reviewers met to discuss and resolve these. The inter-rater reliability of the total score for each paper was 100% as the few disagreements appeared for specific quality indicators and not overall scores. The scores are presented in Table 3.

## Results

### Participant information

Four of the studies were conducted exclusively with participants with profound and multiple learning disabilities (Fenech, 2015; Ockelford et al., 2011; Rushton and Kossyvaki, 2020; Thompson and McFerran, 2015). Three of the studies included participants with profound and multiple learning disabilities alongside participants with other learning disabilities (Bosch et al., 2017; Kossyvaki and Curran, 2020; Wilson and MacDonald, 2019). One study (Wilson and MacDonald, 2019) did not specify the number of participants with profound and multiple learning disabilities. The total number of people identified with profound and multiple learning disabilities within the remaining reviewed studies was 46, with an age range of 5 years to 74 years. Four studies were conducted with child participants or teenage (under 18 years) and three with adults (18+ years). The gender of the participants with profound and multiple learning disabilities was unknown in three of the reviewed studies (Bosch et al., 2017; Ockelford et al., 2011; Wilson and MacDonald, 2019); in the remaining studies there were 13 female and 11 male participants.

All 46 participants were described as having a profound intellectual disability. Additional descriptions of participants included non-verbal, wheelchair user, visual impairment, epilepsy, cerebral palsy, complex needs and autism.

Three studies implemented one-to-one interventions (Bosch et al., 2017; Kossyvaki and Curran, 2020; Thompson and McFerran, 2015). One study (Rushton and Kossyvaki, 2020) delivered the intervention in a 1 facilitator: 2 participants format. The remaining three studies implemented group interventions. The number of participants in each group was unclear for two studies (Fenech, 2015; Wilson and MacDonald (2019). In Ockelford et al. (2011), groups included six or seven participants with profound and multiple learning disabilities.

### Musical Qualities

#### Musical Activity

In five of the seven papers, the intervention which took place was social music activity. Music was used to support the social interaction and behaviours of people with profound and multiple learning disabilities, in either an educational or recreational context. One study used music therapy, which was also used to support the social communication of the participants (Thompson and McFerran, 2015). Music-based interventions were used to support engagement (Fenech 2015; Kossyvaki and Curran, 2020), communicative and social behaviours (Kossyvaki and Curran, 2020; Rushton and Kossyvaki, 2020; Thompson and McFerran, 2015; Wilson and MacDonald, 2019) and individual mood (Bosch et al., 2017). For one study (Ockelford et al., 2011), the intended outcome and measure for the music-based intervention was the development of musical skills.

Participants played an active role in the musical experience in most papers (n=6), in that the participants contributed to the sounds generated or material used. In the music-based intervention described by Bosch et al. (2017), participants took a passive role which included listening to a different pre-determined soundscape.

### Musical elements

Three studies reported interventions which exclusively used live music (Ockelford et al. 2011; Thompson and McFerran, 2015; Wilson and MacDonald, 2019), two pre-recorded music (Bosch et al., 2017; Kossyvaki and Curran, 2020) and two a combination of pre-recorded and live music (Fenech, 2015; Rushton and Kossyvaki, 2020). The musical preferences of participants affected the musical content of four of the interventions. Participants’ preferred songs (Fenech, 2015; Thompson and McFerran, 2015; Wilson and MacDonald, 2019) or ‘personal music preferences’ were used by the facilitators within these music-based intervention sessions (Kossyvaki and Curran, 2020;130).

The structure and content of the intervention sessions varied greatly. Four interventions reported using a structured approach. Two used greeting and ending songs to frame the intervention (Ockelford et al. 2011; Thompson and McFerran, 2015), one described a warm-up component (Wilson and MacDonald, 2019), and another split the intervention into two sections, one-to-one interaction followed by peer-play (Rushton and Kossyvaki, 2020). There were set activities used in the music-based intervention reported by Kossyvaki and Curran (2020), but these were not delivered in a pre-determined order.

A wide variety of musical activity was described within the reviewed studies, these varied in detail and consistency. Four studies provided a detailed framework of pre-developed musical activities and processes which formed the intervention (Bosch et al., 2017; Ockelford et al., 2011; Kossyvaki and Curran, 2020; Rushton and Kossyvaki, 2020). The remaining studies provided descriptions of a range of musical activities which may have been included in the intervention sessions (Fenech, 2015; Thompson and McFerran, 2015; Wilson and MacDonald, 2019); however, the detail of these varied greatly. Singing featured in four of the interventions (Fenech, 2015; Ockelford et al., 2011; Thompson and McFerran, 2015; Wilson and MacDonald, 2019). Improvisation and exploratory musical elements were also described in three of the studies (Kossyvaki and Curran, 2020; Rushton and Kossyvaki, 2020; Thompson and McFerran, 2015).

### Intervention

#### Duration and Frequency

In the reviewed papers, music-based intervention sessions involving people with profound and multiple learning disabilities lasted between 12 minutes and two-hours. The majority (n=5) of interventions lasted between 20 and 45 minutes, with one intervention shorter in duration, lasting 12 minutes (Kossyvaki and Curran, 2020), and one significantly longer, lasting two-hours (Wilson and MacDonald, 2019). For studies in which the period of intervention was known, the timescale was between five-weeks and six-months in duration. Sessions were delivered on a once or twice weekly basis. In three of the studies (Bosch et al., 2017; Fenech, 2015; Thompson and McFerran, 2015), it was not possible to ascertain the frequency or regularity of the interventions. Participant absence was recorded in five of the reviewed studies (Ockelford et al., 2011; Kossyvaki and Curran, 2020; Rushton and Kossyvaki, 2020; Thompson and McFerran, 2015; Wilson and MacDonald, 2019).

### Equipment or materials

Five studies reported participants having access to a range of untuned percussion instruments as part of the intervention (Fenech, 2015; Ockelford et al., 2011; Rushton and Kossyvaki, 2020; Thompson and McFerran, 2015; Wilson and MacDonald 2019), two studies used music-technology (Bosch et al., 2017; Kossyvaki and Curran, 2020) as the principle musical element of the intervention, without the use of musical instruments. Except for Kossyvaki and Curran (2020), which used a pre-programmed digital interface, called ‘Cosmo’ (Cosmo by Filisia, 2017), the instruments used with and by participants in all the interventions are likely to be available in most music-classrooms or specialist settings.

### Facilitator

In six of the studies facilitators who delivered the intervention had a specialist music role or previous musical training. These included music therapist (Thompson and McFerran, 2015), music teacher (Ockelford et al., 2011; Kossyvaki and Curran, 2020), music specialist (Rushton and Kossyvaki, 2020; Wilson and MacDonald, 2019), and disk-jockey (DJ) (Fenech, 2015). One of the studies did not include facilitators who held a specialist music role (Bosch et al., 2017); this intervention was a listening-based intervention in which pre-recorded music and sound was played to the participants. In most studies (n=5), it is unclear whether the role and interactions of the facilitators varied throughout the intervention. The process of establishing, building and developing relationships, between facilitator and participant, is highlighted in Kossyvaki and Curran (2020) and Thompson and McFerran (2015); however, there is little discussion of the impact this may have had on the effectiveness and outcomes of the interventions.

### Setting

The majority (n=6) of the interventions took place in the participants’ typical setting. This was either a school or care setting depending on the age of the participants. Four studies (Bosch et al., 2017; Fenech, 2015; Kossyvaki and Curran, 2020; Rushton and Kossyvaki, 2020) reported using a dedicated space within the participants’ typical setting to deliver the intervention sessions.

In one study (Wilson and MacDonald, 2019), the music-based intervention sessions were reported to partially take place in a community rehearsal setting; however, the paper does not say specifically whether the group of participants with profound and multiple learning disabilities attended sessions at the community rehearsal setting.

### Intended outcomes

There were a variety of intended outcomes which were measured during the music-based interventions. These included communicative and social behaviours (n=4) (Kossyvaki and Curran, 2020; Rushton and Kossyvaki, 2020; Thompson and McFerran, 2015; Wilson and MacDonald, 2019), engagement (n=2) (Fenech, 2015; Kossyvaki and Curran, 2020), mood (Bosch et al., 2017) and musical reactivity (Ockelford et al. 2011). For most studies (n=5), these outcomes were measured for individual participants, using the frequency of observable participant behaviours. Fenech (2015) measured engagement level outcomes for the group, and for Wilson and MacDonald (2019), there is minimal comment on the discrete outcomes of the sessions for participants with profound and multiple learning disabilities.

### Effectiveness

Five studies reported positive findings (Bosch et al., 2017; Fenech, 2015; Ockelford et al., 2011; Rushton and Kossyvaki, 2020; Wilson and MacDonald, 2019) and one study reported mixed results. Kossyvaki and Curran (2020) found fluctuating levels of engagement and medium to high levels of disengagement during the intervention. The study also reported an increased in awareness, anticipation and initiation by the participant, which setting staff claimed was transferred to other contexts. One study (Thompson and McFerran, 2015) reported no difference in the outcomes of the music-based intervention from the control condition of toy play. However, whilst the number of communicative acts was similar in both conditions, the researchers reported higher scores in social responses, and fewer ‘reject/protest’ behaviours during the music sessions, and highlighted music as a motivating factor within relationships for people with profound and multiple learning disabilities within their conclusion.

A range of data collection materials were used to measure the outcomes of the interventions. These included checklists and frameworks designed by the authors (n=3) (Bosch et al., 2017; Kossyvaki and Curran, 2020; Ockelford et al., 2011). Two studies used simplified versions of pre-existing observation checklists (Rushton and Kossyvaki, 2020; Thompson and McFerran, 2015). Fenech (2015) used a pre-existing data collection framework, the Individual Child Engagement Record (ICER) (Kishida and Kemp, 2006), originally developed for children with intellectual disabilities.

Two studies (Kossyvaki and Curran, 2020; Rushton and Kossyvaki, 2020) further explored participant outcomes through interviews with supporting staff. Wilson and MacDonald (2019) collected qualitative data through interviews with family members and unstructured observations of the intervention sessions.

## Discussion

This systematic review of music-based interventions, involving individuals with profound and multiple learning disabilities revealed a limited number of diverse and disparate studies. A review of music-based interventions for the population of people with profound and multiple learning disabilities has not previously been conducted. The discussion which follows will be of interest to researchers, educators and practitioners working with individuals with profound and multiple learning disabilities.

### Participants

The participants of the reviewed interventions included both children and adults. Although most interventions (n=4) focused on participants under 18 years old, some interventions (Bosch et al., 2017; Fenech, 2015; Wilson and MacDonald, 2019) included adults with profound and multiple learning disabilities. This is in slight disagreement with intervention research for participants with other disabilities which found that most studies included young children and highlighted the need for more research to be conducted in adolescents and adults (Kossyvaki and Papoudi, 2016; Levy and Perry, 2011). Interestingly, the delivery of interventions with adult participants was likely to also include other participants with differing levels of intellectual impairment and disability.

### Musical Qualities

#### Musical activity

The musical activity which took place in most interventions was social (n=5). One study used music therapy, although the focus of this study was also communication, and one music education. The prominence of social musical activity within the interventions is supported by literature which highlights the role of music in supporting the development of early communication and interaction skills of people with profound and multiple learning disabilities (Corke, 2012; Imray and Hinchcliffe, 2014). The focus of the interventions to develop engagement and social communication is consistent with reviews of music-based interventions involving participants with other disabilities (James et al., 2015; Simpson and Keen, 2011).

Music therapy, used in the intervention reported by Thompson and McFerran (2015), is a limited resource as it requires a specialist trained music therapist. Few people with profound and multiple learning disabilities are likely to have regular access to a music therapist (Imray and Hinchcliffe, 2014; Ockelford, 2008); therefore, the accessibility of the intervention may be compromised. The usefulness of discretely measuring musical skill development, as the outcome in Ockelford et al. (2011) is questionable. The analysis of specific subject-based progress, such as musical skills, is no longer expected to be measured for individuals with profound and multiple learning disabilities in England, UK (Rochford Review, 2016). Whilst people with profound and multiple learning disabilities may develop skills for music making, it is considered that these individuals are at the earliest stage of developmental and therefore unlikely to develop subject-specific skills (Imray and Hinchcliffe, 2014).

Most interventions (n=6) described the participants as playing an active role in the musical experience, in which they contributed to their musical environment through singing, instrumental playing or controlling the musical sounds. This active role supports the current literature, and wider shifting perceptions of people with profound and multiple learning disabilities, from being passive individuals to active contributors within their interactions and relationships (Nind & Strnadová, 2020).

### Intervention

#### Structure and content

Many studies failed to provide detailed descriptions of the musical content of the interventions; therefore, they are not likely to be replicable. Some interventions (n=4) used structure and routine. Predictability and routine can facilitate participants with profound and multiple learning disabilities to feel more secure and provided opportunities for individuals to anticipate events or actions. This approach replicates the routine and structure many individuals with learning disabilities experience and require to support their navigation of daily life (Doukas et al., 2017).

Singing was described in most (n=4) of the studies. Improvisation and exploratory musical elements were described in three of the studies (Kossyvaki and Curran, 2020; Rushton and Kossyvaki, 2020; Thompson and McFerran, 2015). The frequent use of these two musical elements, song and improvisation, is consistent with the findings of reviews of music-based interventions for participants with other disabilities (James et al., 2015; Simpson and Keen, 2011).

### Facilitator and setting

Most of the studies (n=6) used facilitators with specialist music experience to deliver the interventions. This is notable, as access to specialist music facilitators, educators or therapists is a limited resource (Imray and Hinchcliffe, 2014; Ockelford, 2008). The use of music specialists to deliver the intervention may impact on ecological validity of most of the interventions, and feasibility of implementing the intervention in some settings. Further, it may dissuade non-music specialist teaching staff from employing the intervention in their setting.

Five of the studies included staff already working with the participants to contribute to the content of intervention and data analysis (Kossyvaki and Curran, 2020; Thompson and McFerran, 2015) or deliver and evaluate the intervention (Bosch et al., 2017; Ockelford et al., 2011; Rushton and Kossyvaki, 2020). The familiarity and knowledge developed within previously established relationships is likely to support the implementation, interpretation, and analysis of the often idiosyncratic behaviours and responses of participants with profound and multiple learning disabilities (McFerran and Shoemark, 2013; Simmons, 2021). Furthermore, as facilitators became more familiar with working with the individuals with profound and multiple learning disabilities (Kossyvaki and Curran, 2020; Thompson and McFerran, 2015), and/or the intervention, (Bosch et al., 2017; Ockelford et al., 2011; Rushton and Kossyvaki, 2020) their roles and interactions may have altered. This added variable, of increased familiarity, is likely to have affected the implementation, effectiveness and outcomes of the interventions.

Most (n=6) studies took place in real-world settings. Whilst this may have compromised the external validity and generalisability of the findings (Harackiewicz and Barron, 2004), the ecological validity of the interventions was strong.

### Duration and frequency

The majority (n=5) of the interventions lasted between 20 and 45 minutes; however, there was great variability in the duration, with the shortest being 12 minutes and the longest two-hours. Interventions were delivered on a weekly or twice weekly basis and were implemented for between 5 weeks and six months. The duration and frequency of the intervention sessions seem viable to be delivered within the weekly schedules of people with profound and multiple learning disabilities, whether in educational or care settings. Participant absence, which is likely due to the complex health needs of people with profound and multiple learning disabilities, was recorded in five of the reviewed studies. Absences or irregular attendance is more likely for individuals with profound and multiple learning disabilities (Public Health England, 2019) and this should be considered when designing and implementing interventions for this population, to ensure there is a sufficient period of access to the intervention programme.

### Equipment or materials

Most of the studies within this review (n=5) described using a range of untuned percussion instruments, and pitched instruments such as guitar, keyboard and Boom Whackers, which are likely to be found in most school, and some care, settings as part of the intervention. In contrast to the specialist ‘Cosmo’ digital interface used in Kossyvaki and Curran (2020), the use of these commonly found instruments within most interventions increases their ecological validity. The use of readily available materials in typically occurring settings to support the ecological validity of an intervention has been highlighted (Ledford et al., 2016).

### Effectiveness

Five of the studies reported positive findings, whereas two reported mixed results. Kossyvaki and Curran (2020) found inconsistent levels of engagement, whilst reporting increased awareness and attentiveness. Thompson and McFerran (2015) also found inconsistencies across the potentially communicative acts they observed. Three studies commented on the limited generalisability of the findings (Bosch et al., 2017; Kossyvaki and Curran, 2020; Thompson and McFerran, 2015) due to the small sample size and idiosyncratic nature of the population. The effectiveness of most interventions is questionable as the individuals facilitating the intervention were also involved in its evaluation. It is unclear, from the identified studies, if the social and behavioural changes reported are attributed to participant development, as a result of the intervention, or whether this occurred because of the altered behaviour, interactions and familiarity of the facilitators. Furthermore, the frameworks used may have compromised the claims of effectiveness.

### Implications for future research and practice

People with profound and multiple learning disabilities are a heterogenous population (Bellamy et al., 2010). It appears that the research which has been undertaken in relation to music-based interventions is as varied as the population itself. The intended outcomes and measurement tools varied across the studies, and the data collection tools were frequently designed or adapted for the purpose of the individual intervention, which might have compromised the effectiveness reported. Developing, piloting and validating data collections tools, which are fit for purpose when researching people with profound and multiple learning disabilities, may further improve the validity of the findings of future research.

Rigorous experimental studies such as randomised control trials may not be suitable for research designs involving people with profound and multiple learning disabilities. Due to the heterogeneity and low prevalence of this population, it is unlikely that large numbers of participants with similar characteristics can be found. However, extended studies which include a baselining period, or a control environment, would increase the reliability of the study and claims of effectiveness.

Musical activity is a creative and malleable process, often resulting in highly individualised interactions and responses. This, and the real-world implementation of the interventions, may have compromised the documentation of the procedures used within the reviewed interventions. The procedures described for few interventions were detailed enough to be replicated (Bosch et al., 2017; Kossyvaki and Curran, 2020; Ockelford et al., 2011). Replicability of other interventions was compromised due to a lack of detail in either the musical content used, or the intervention procedures employed by the facilitator. Whilst musical interactions are likely to be fluid and variable processes (Hallam and MacDonald, 2013) robust and detailed intervention procedures may increase the likelihood and success of practitioners implementing the interventions within their own settings. Further, high quality, robust research may lead to better understanding of how music can be used, and perhaps more importantly, the effects and personal preferences of music for individuals with profound and multiple learning disabilities. Providing musical experiences with understanding of an individuals’ preference and choice can create engaging activities and leisure time for people with profound and multiple learning disabilities (McFerran and Shoemark, 2013; Rushton and Kossyvaki, 2020) enhancing their quality of life (Nieuwenhuijse et al., 2020). This knowledge, choice and opportunity is particularly important due to the large amounts of spare, unstructured time people with profound and multiple learning disabilities are likely to experience in adulthood (Imray and Hinchcliffe, 2014).

## Conclusion

Due to the diversity of the population of people with profound and multiple learning disabilities, and more specifically those included within the reviewed papers, a direct comparison of the studies is limited. The review of the literature is limited as it included studies written only in English, between January 2010 and April 2021. Adopting the biopsychosocial model of disability, the reviewers aimed to consider the interventions in relation to the participants, their environment, and the approaches of others. Using this model acknowledged the potential biological, physiological, and cognitive state of people with profound and multiple learning disabilities, whilst realising the further disabling physical, attitudinal, and social barriers they encounter daily. The positionality of the researchers has likely influenced the focus of discussion. Data collected in all studies relied heavily on the facilitator and/or researcher interpreting and categorising observable behaviours of the participants.

The experimental nature of the interventions reviewed within this study highlights a need to increase the quantity and quality of research undertaken to better understand how music can be used in a high quality and effective way with people with profound and multiple learning disabilities. The development of validated data collection tools, which are objective, robust, and useful may further support claims of effectiveness when using music with individuals with profound and multiple learning disabilities. Additionally, increased collaboration between music specialists and setting staff, non-music specialists who work daily with individuals with profound and multiple learning disabilities, to develop effective music-based interventions may increase the ecological validity and access to music-based interventions for people with profound and multiple learning disabilities. Effective development of these interventions must be congruent with robust research design and clear dissemination of the protocol and procedures involved to ensure replicability, whilst also being flexible to the musical preferences and individuality of those taking part.
